# Alternol, a natural compound, exerts an anti‐tumour effect on osteosarcoma by modulating of STAT3 and ROS/MAPK signalling pathways

**DOI:** 10.1111/jcmm.12957

**Published:** 2016-09-14

**Authors:** Dongqing Zuo, Zifei Zhou, Hongsheng Wang, Tao Zhang, Jie Zang, Fei Yin, Wei Sun, Jiepeng Chen, Lili Duan, Jing Xu, Zhuoying Wang, Chongren Wang, Binhui Lin, Zeze Fu, Yuxin Liao, Suoyuan Li, Mengxiong Sun, Yingqi Hua, Longpo Zheng, Zhengdong Cai

**Affiliations:** ^1^ Department of OrthopedicsShanghai Tenth People's HospitalSchool of MedicineTongji UniversityShanghaiChina; ^2^ Department of OrthopaedicsShanghai First People's HospitalShanghai Jiao Tong UniversityShanghaiChina; ^3^ Department of OrthopaedicsShanghai East HospitalSchool of MedicineTongji UniversityShanghaiChina; ^4^ Department of OrthopaedicsYangpu HospitalSchool of MedicineTongji UniversityShanghaiChina; ^5^ Musculoskeletal Tumor CenterPeople's HospitalPeking UniversityBeijingChina; ^6^ Strand Biotechnology Institute of ResearchShantouChina

**Keywords:** osteosarcoma, alternol, chemotherapy, reactive oxygen species, MAPKs, STAT3

## Abstract

Osteosarcoma (OS) is the most frequent primary malignant bone tumour. Alternol, a novel compound purified from microbial fermentation products exerts anti‐tumour effects across several cancer types. The effect of alternol on human OS remains to be elucidated. We first evaluated the anti‐tumour effect of alternol in several human OS cell lines *in vitro* and investigated its underlying mechanism. Alternol inhibited OS cell proliferation, migration and induced caspase‐dependent apoptosis, G2/M cell cycle arrest in a dose and time‐dependent manner. Moreover, alternol treatment inhibited signal transducer and activator of transcription‐3 (STAT3) phosphorylation in 143B and MG63 human OS cells, as evaluated using a STAT3‐dependent dual luciferase reporter system. Exposure to alternol resulted in excessive reactive oxygen species (ROS) generation and Jun amino‐terminal kinases (JNK), extracellular signal‐regulated kinases (ERK1/2) and p38 activation. Furthermore, alternol‐induced cell death was significantly restored in the presence of the ROS scavenger, *N*‐acetyl‐l‐cysteine (NAC) or a caspase inhibitor Z‐VAD‐FMK. NAC also prevented G2/M phase arrest and phosphorylation of mitogen‐activated protein kinases (MAPK), but did not reverse STAT3 inactivation. Finally, alternol suppressed tumour growth *in vivo* in the nude mouse OS tibia orthotopic model. Immunohistochemistry revealed that alternol treatment resulted in down‐regulation of phosph‐STAT3 Tyr705 and up‐regulation of cleaved caspase‐3 and phosph‐SAPK (Stress‐activated protein kinases)/JNK expression. Taken together, our results reveal that alternol suppresses cell proliferation, migration and induces apoptosis, cell cycle arrest by modulating of ROS‐dependent MAPK and STAT3 signalling pathways in human OS cells. Therefore, alternol is a promising candidate for developing anti‐tumour drugs target OS.

## Introduction

Osteosarcoma is the most common primary malignant bone tumour, accounting for approximately 60% of all bone sarcoma, OS has a rare worldwide incidence of approximately one to three cases annually per million, and 5‐year overall survival is approximately 50–70%^1^. Current therapies include surgical resection and combination neo‐adjuvant chemotherapy (doxorubicin and cisplatin with or without methotrexate) cure less than 70% of patients 2. However, the survival of patients with metastatic or relapsed OS has remained virtually unchanged over the past 30 years, with an overall 5‐year survival rate of approximately 20%. Systematic toxicity due to non‐specific targeting of human normal and cancer cells also impairs the immune function of cancer patient, and thus contributing to cancer deterioration. Therefore, new therapies with a lower working dose and improved targeting are urgently needed.

Paclitaxel is one of the most commonly used taxane chemotherapeutics in cancer chemotherapy, particularly in breast cancer 3, ovarian cancer 4 and non‐small cell lung carcinoma 5 treatment. Paclitaxel is also effective in head‐and‐neck cancers 6, oesophageal cancer 7 and recurrent non‐Hodgkin's lymphoma 8. As a new taxane chemotherapeutic, alternol has been isolated from the fermentation product of a novel microorganism mutant strain *Alternaria alternata var. monosporus*, which was obtained from the bark of yew trees in Kunming, China 9. Alternol can induce cell cycle arrest and apoptotic cell death and inhibit epithelial‐to‐mesenchymal transition 10 in human cancer cell lines. Yeung *et al*. 11 observed that alternol preferentially kills prostate cancer cells over nonmalignant prostatic epithelial cells, indicating a potential benefit for patients with prostate cancer. However, the effect of alternol in bone sarcoma remains unclear.

Signal transducer and activator of transcription‐3, is primarily activated and mediated by interleukin‐6 (IL‐6) family cytokine receptor‐associated Janus kinase (JAKs), and regulate the transcriptional activation of anti‐apoptotic and proliferative gene products, such as cyclins, cyclin‐dependent kinases (Cdk) and survivin 12. Paradoxically, STAT3 signalling activation induces cell death under some conditions, such as lysosomal pathway induction in the involuting mammary gland model 13, 14. Other researchers found that inactivation of the STAT3 pathway decreases tumour growth and invasion through its targeted gene in both *in vitro* and *in vivo* studies, including in OS and gastric cancer 15, 16. STAT3 function has increasingly become focus of anti‐tumour research.

Reactive oxygen species are chemically oxygen‐containing molecules such as peroxides, superoxide, hydroxyl radical and singlet oxygen 17. Reactive oxygen species are formed as a byproduct of the normal metabolism of oxygen and play important roles in cell signalling and homeostasis. Under normal conditions, mitochondria trigger redox signalling in cells *via* the release of ROS from the electron transport chain. Under pathophysiological conditions, ROS generation from the mitochondria can also contribute to the initiation of cancer and amplification of the tumour cell phenotype 18. However, mitochondrial ROS may also make tumour cells vulnerable to therapies that further decrease their ability to regulate redox homeostasis, introducing opportunities for novel effective anti‐tumour therapies 19.

In this study, we investigated the anti‐proliferation, anti‐migration and pro‐apoptotic role of alternol in several human OS cell lines *in vitro* and in nude mice bearing tibia tumour, we also explored the underlying molecular interaction in human OS cell to fully understand its anti‐tumour mechanisms.

## Materials and methods

### Cell lines and culture

Human OS cell lines 143B, KRIB, MG63, U2OS were obtained from American Type Culture Collection. All cells were cultured in high glucose DMEM (DMEM‐h; Thermo, Waltham, MA, USA) supplemented with 10% foetal bovine serum (Thermo), 100 U/ml penicillin and 100 μg/ml streptomycin (Thermo) in a humidified incubator at 37°C in 5% CO_2_.

### Drugs and antibodies

**Figure 1:**
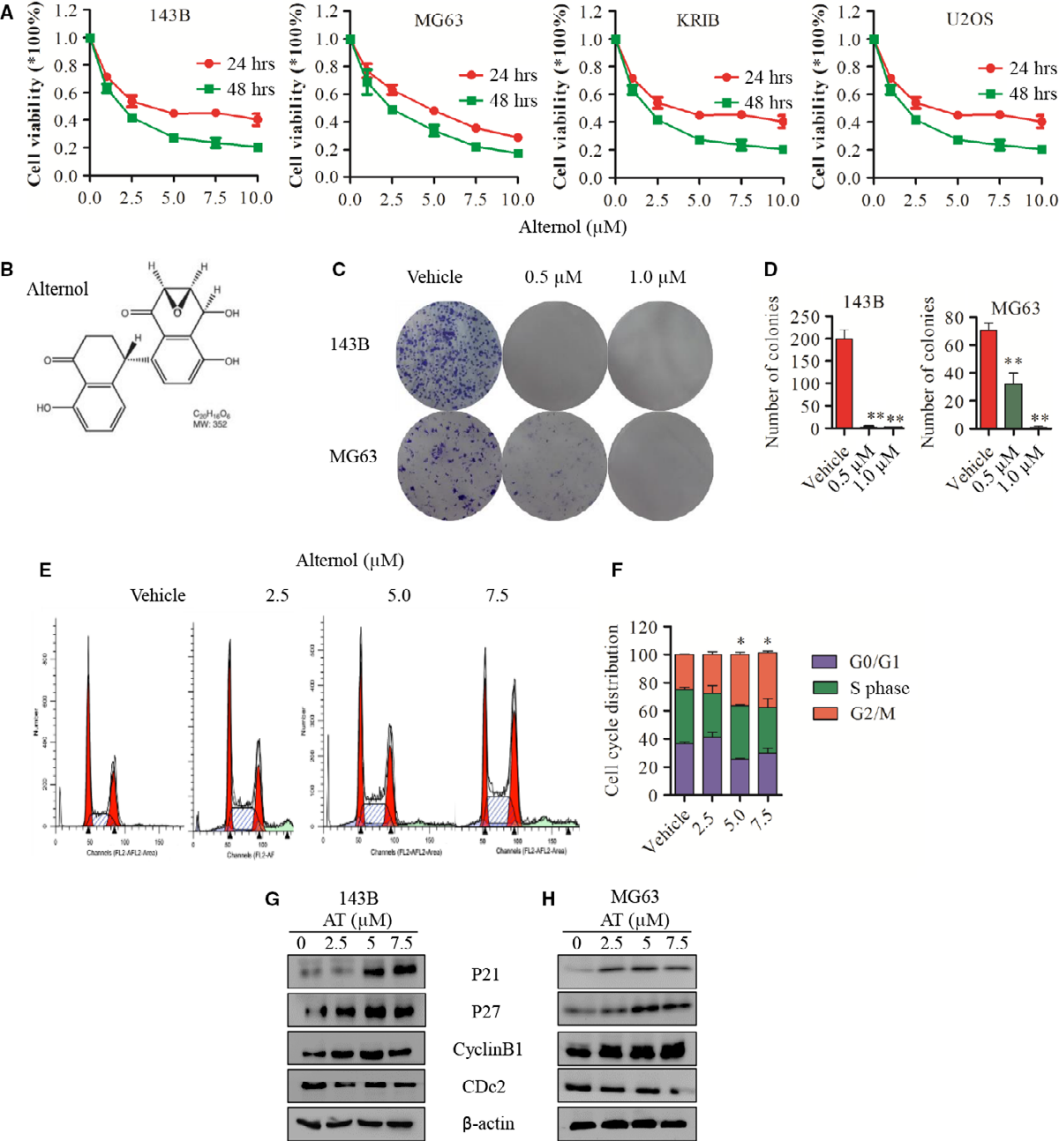
Alternol inhibits OS cells proliferation and induces G2/M cell cycle arrest in human OS cells. (**A**) Human osteosarcoma cell line 143B, MG63, U2OS, KRIB cells were treated with vehicle (0.1% DMSO) or alternol (2.5, 5.0 and 7.5 μM) for 24 or 48 hrs, cell viability was measured by CCK8 assay. (**B**) Chemical structure of alternol. (**C** and **D**) Cell colony formation of 143B and MG63 treated with vehicle or alternol. (**E**) 143B and MG63 were treated with vehicle or alternol (2.5 and 5.0 μM) for 12 hrs, cell cycle was analysed using flow cytometry. (**F**) Cell cycle distribution presented as the mean ± S.D. from three independent experiments. (**G** and **H**) 143B and MG63 were treated with vehicle or alternol (2.5 and 5.0 μM) for 12 hrs, cell cycle proteins p21, p27, cyclinB1 and CDc2 expression were determined by western blot. *P < 0.05 *versus* with vehicle control, **P < 0.01 *versus* with vehicle control.

Alternol (99.9% purity) is a kind gift from Strand Biotech Co. Shantou, China and its structural scheme is shown in Figure 1B. It was dissolved in dimethyl sulfoxide (DMSO) as a 10 mmol/l stock solution stored from light in aliquot package in −20°C. The working concentrations used for different experiments were prepared by diluting the stock solution with DMEM‐h. The antibodies used for western blot were as follows: rabbit anti‐actin (Santa Cruz Biotechnology, Santa Cruz, CA, USA), anti‐caspase‐3, anti‐caspase‐8, anti‐Bcl‐xl, anti‐PARP anti‐p27, anti‐p21, anti‐CyclinB1, anti‐CyclinA2, anti‐CyclinD1, anti‐CDc2, anti‐SAPK/JNK, anti‐phosph‐SAPK/JNK (Tyr183/185), anti‐p38MAPK, anti‐phosph‐p38MAPK (Tyr180/182), anti‐ERK1/2, anti‐phosph‐ERK1/2 (Tyr202/204), anti‐STAT3, anti‐phosph‐STAT3 (Tyr705), anti‐JAK2, anti‐phosph‐JAK2 (Tyr1007/1008), anti‐Src, anti‐phosph‐Src (Tyr416) (Cell Signaling Technology Inc., Danvers, MA, USA), caspase3 inhibitor Z‐VAD‐FMK, SAPK/JNK‐specific inhibitor SP600125, p38MAPK inhibitor SB203580 (Selleck, Selleckchemo Houston, TX, USA), ROS inhibitor antioxidant NAC (Beyotime, Shanghai, China), human IL‐6 (Sigma‐Aldrich, Inc., St. Louis, MO, USA).

### CCK8 cell viability assay

Cells were seeded into 96‐well plates and treated with alternol at indicated concentrations for 24/48 hrs. Cells incubated with 0.1% DMSO DMEM‐h were regarded as vehicle control group. After 24/48 hrs incubation, 20 μl CCK8 (5 mg/ml; Dojindo Molecular Technologies, Inc., Kumamoto Techno Research Park Tabaru, Mashikimachi, kamimashiki gun, Kumamoto, JAPAN) was added into each well for another 4 hrs incubation 37°C following the manufacturer's protocol. After that, the absorbance was then measured using a model ELX800 Micro Plate Reader (Bio‐Tec Instruments, Inc., Bio‐Tek Winooski, VT, USA) at 490 nm. EXCEL FORCAST (x, known‐y′s known‐x′s) was used to predict the drug IC50 level 20. Three independent experiments were carried out in triplicate.

### Cell colony formation assay

Cells were seeded in six‐well plates (500/well) and dispersed evenly by shaking the dishes slightly. After attachment, cells were treated with vehicle or alternol (0.5 and 1.0 μM) for about 10–14 days when the cells grow to visible colonies. The medium was discarded and the cells were washed with PBS twice. After being fixed with 4% paraformaldehyde for 20 min., washed with PBS, the cells were stained with 0.1% crystal violet for 15 min. and washed with PBS. The colonies with more than 50 cells were counted under an ordinary optical microscope.

### Flow cytometry analysis

143B and MG63 OS cells were cultured in six‐well plates (2.5 × 10^5^/well) and treated as indicated for 12 hrs. Cell cycle and apoptosis proceeded as we previously described 21. Apoptosis was detected by FCM and analysed using BD Cellquest software (BD Biosciences, San Jose, CA, USA). Dual parameter dot plots combining Annexin V‐FITC/PI revealed live cells in the lower left (Annexin V−/PI−), early apoptotic cells in the lower right (Annexin V+/PI−), late apoptotic cells in the upper right (Annexin V−/PI+), and necrotic cells in the upper left (Annexin V+/PI+) quadrant.

### Wound‐healing assay

143B and MG63 OS cells were seeded in 12‐well plates and when growing into full confluence, a ‘wound’ was scratched by a sterile 100 μl pipette tip. Fresh medium containing different concentrations of alternol was subsequently added. After 12 hrs, cells were fixed with 4% paraformaldehyde, and images were obtained by an inverted microscope (Olympus, Nishishinjuku Shinjuku‐Ku, Tokyo, Japan). Migrated distance was measured by Image J software (National Institutes of Health, MD, USA). The independent experiments were carried out in triplicate.

### Transwell invasion assay

143B and MG63 OS cells were suspended at 5 × 10^4^ cells in 100 μl medium with or without indicated concentrations of alternol and added to each transwell insert. 500 μl of growth medium was placed in each bottom well. 12 hrs post seeding, invaded cells in the lower side of the insert were fixed with 4% paraformaldehyde and stained with 0.1% crystal violet. Images were acquired by an inverted microscope (Olympus) and invaded cells were counted manually. Three independent experiments were carried out in triplicate.

### Measurement of ROS

Generation of intracellular ROS was measured by using DCFH‐DA staining (Beyotime). In brief, cells were plated at a density of 2.5 × 10^5^/well in six‐well plates and exposed to vehicle or alternol (2.5 and 5.0 μM) for 12 hrs. Then, the cells were trypsinized and incubated with DCFH‐DA (10 μM) for 30 min. at 37°C in dark. Reactive oxygen species generation was determined by confocal microscopy and flow cytometer.

### Hoechst assay

The Hoechst assay was used to evaluate the apoptotic cell death to alternol treatment. 143B and MG63 OS cells were seeded on coverslips at a density of 2.5 × 10^5^ cells per well in a six‐well plates and exposed to vehicle or alternol (5.0 μM) for 12 hrs. after being fixed with 4% paraformaldehyde for 20 min., a One Step Hoechst Kit was uesd (Beyotime), the cells were observed under fluorescence microscope for apoptosis.

### Immunofluorescence assay

Cells seeded on coverslips were exposed to suggested treatments for 12 hrs, then fixed with 4% paraformaldehyde and permeabilized with 0.1% Triton X‐100 in PBS. Samples were blocked with 1% bovine serum albumin for 30 min. followed by incubation with indicated primary antibodies at 4°C overnight. After three washes, cells were probed with Alexa Fluor 488 secondary antibody for 1 hr at room temperature. The nuclei were stained by 4′,6‐diamidino‐2‐phenylindole (DAPI). Images were acquired with a fluorescence microscope (Leica, Wetzlar, Germany).

### Western blot assay

Cells were washed twice with cold 1× PBS solution, lysed with RIPA lysis Buffer (Beyotime) with phosphorylase and protease inhibitor (Beyotime). Protein concentrations were determined with Standard BCA Protein Assay Kit (Beyotime). Equivalent amounts of total protein (30–60 μg) were boiled and electrophoretically separated with 10% or 15% polyacrylamide gel at 80–120 V. Proteins were transferred to a nitrocellulose filter membrane. Membranes were blocked for 60 min. with 5% milk solutions prepared in PBST (Phosphate Buffered Saline with 0.1% Tween^®^ 20), incubated overnight at 4°C with 1:1000 dilutions of the primary antibodies, washed three times for 10 min. each time with Tween 20 (1:1000 dilution)‐PBS, incubated for 1 hr with the appropriate peroxidase conjugated secondary antibody (1:5000 dilution). Membranes were washed with Tween 20‐PBS three times for 10 min. each and were developed using the Odyssey two colour infrared laser imaging system. The signal generated by B‐actin was used as an internal control.

### STAT3 luciferase reporter gene assay

143B and MG63 cells were seeded into 12‐well plates at a density of 5 × 10^4^ cells per well prior to transfection. Cells were cotransfected with pGMSTAT3‐Lu and pRL‐SV40 (a plasmid encoding Renilla luciferase) (YeasenBiotech, Shanghai, China) using Lipo2000 transfection reagent (Invitrogen, Grand Island, NY, USA). Twenty‐four hours after transfection, the cells were treated with 5.0 μM alternol for an additional 12 hrs and a cell lysate was prepared with reporter lysis buffer. After mixing the cell lysates with luciferase substrate (E1910; Promega, Madison, WI, USA), the luciferase activity was measured using a luminometer. The luciferase activities of STAT3 in the vehicle and treatment groups were normalized by Renilla luciferase activity.

### Nude mouse tibia orthotopic tumour model

Four‐week‐old female BABL/c nude mice were housed under standard conditions with a 12‐h light‐dark cycle and fed with sufficient water and food. All the animal procedures were performed in accordance with a protocol approved by the Animal Care and use Committee of Shanghai General Hospital and Shanghai Jiaotong University. A total of 10^6^ 143B cells cell suspension in 20 μl of PBS was injected into the right tibia medullary cavity to establish an orthotopic OS model using a 25 gauge needle and a 100 μl syringe. Two weeks after injection, the mice were randomly allocated to 5 mg/kg alternol group (*n* = 5), 10 mg/kg alternol group (*n* = 5), vehicle (DMSO) group (*n* = 4). Each mouse in alternol group received weight‐based alternol dosage by intraperitoneal (ip) injection every 3 days. The vehicle group mice were injected with 100 μl PBS with 10% DMSO the same way. Tibia tumour volume and mouse bw were measured before injection using the formula (volume = length × width × width × 0.5) 22. After 7–10 continuous injections in the three groups, the mice were killed. Tumours were dissected and stored in liquid nitrogen or fixed in formalin for further analysis.

### Immunohistochemistry staining

Tumour samples were sliced and fixed with 10% formalin overnight. Specimens were embedded in paraffin and sliced to 4‐mm‐thick sections before light microscopy evaluation. Expression of cleaved caspase3, phosph‐JNK and phosph‐STAT3 was evaluated by Immunohistochemistry (IHC). The intensity of the staining was scored as 1 (negative), 2 (weakly positive), 3 (moderately positive) or 4 (strongly positive). The extent of the staining was categorized as 1 (1–25% stained cells), 2 (26–50%), 3 (51–75%) or 4 (76–100%). The final staining score was the product of the intensity and the extent scores. Images of five random fields from each specimen were used for quantitative analysis.

### Statistical analysis

Statistical analysis was performed with GraphPad Prism 5 (La Jolla, CA, USA). All measurement data are expressed as the mean ± S.D. Comparisons were made between groups using Student's *t*‐test and two‐way anova. *P* < 0.05 was considered statistically significant.

## Results

### Alternol inhibits OS cell proliferation and induces cell cycle arrest

We first evaluated the cytotoxicity of alternol in OS cells. Cells were treated with different concentration of alternol for 24 and 48 hrs. The IC50 level of 143B, MG63, KRIB and U2OS was 4.9, 5.0, 5.9 and 12.08 μΜ, respectively, after 24 hrs alternol treatment (Fig. 1A). Cell colony formation was then evaluated. Significantly fewer colonies were observed after treatment of 143B and MG63 OS cells with 0.5 and 1.0 μM alternol, confirming the inhibition of proliferation (Fig. 1C and D). These results demonstrated that alternol inhibits the proliferation of OS cells in a dose‐ and time‐dependent manner.

We next evaluated changes in the cell cycle after alternol treatment in OS cells. The cell cycle was inhibited in G2/M phase after 12 hrs of alternol treatment, as assessed by flow cytometry (Fig. 1E). To further elucidate the mechanism of cell cycle arrest, we analysed the cell cycle‐regulated proteins expression by western blot and observed that alternol up‐regulated p21, p27, cyclinB1 expression but down‐regulated the expression of Cdc2 and cyclinD1 (Fig. 1F and G). These data suggest that alternol induces G2/M cell cycle by regulating cell cycle protein in human OS cells.

### Alternol induces caspase‐dependent cell apoptosis in 143B and MG63 human OS cells in a time‐ and dose‐dependent manner

**Figure 2:**
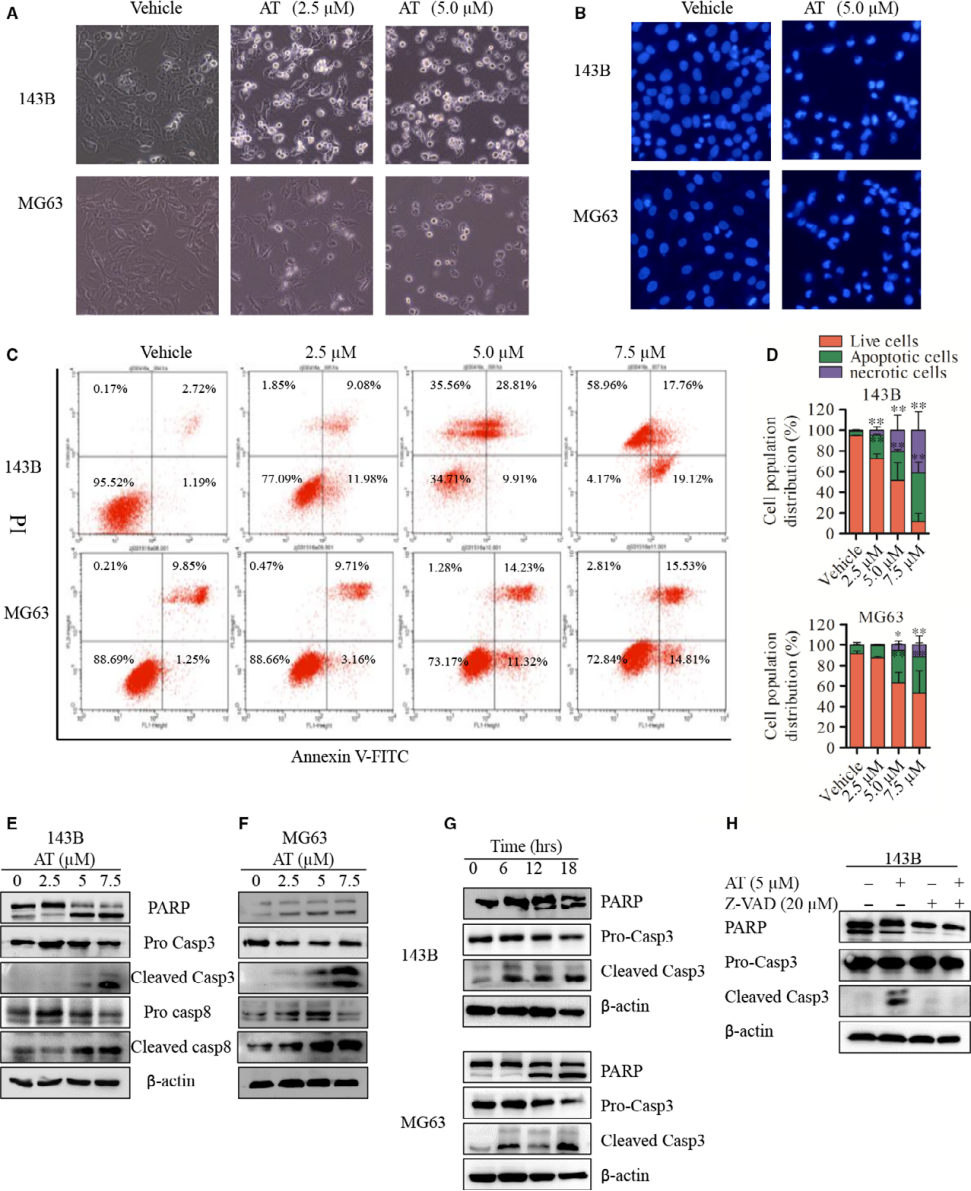
Alternol induces caspase‐dependent cell apoptosis in 143B and MG63 human OS cells in a time‐ and dose‐dependent manner. (**A**) 143B and MG63 OS cells were treated with vehicle or indicated alternol (2.5 and 5.0 μM) for 12 hrs, cell morphology were observed by inverted microscope. (**B**) 143B and MG63 OS cells were treated with vehicle or indicated alternol (5.0 μM) for 12 hrs, cell apoptotic morphological changes were evaluated by fluorescence microscopy using Hoechst 33342. (**C** and **D**) Cell apoptosis index was determined by flow cytometry by Annexin V‐FITC and PI staining after vehicle or alternol treatment for 12 hrs, data are representative of three independent experiments. (**E**–**G**) 143B and MG63 cells were treated with indicated concentrations of alternol for 12 hrs or incubated with alternol (5 μM) for different treatment time (0, 6, 12, 18 hrs). The expressions of cleaved PARP, caspase‐3, ‐8 were determined by western blot. (**H**) 143B cells were treated with vehicle or alternol (5 μM) with presence or absence of caspase inhibitor Z‐VAD (20 μM), the expressions of cleaved PARP, caspase‐3 were determined by western blot. *P < 0.05 *versus* with vehicle control, **P < 0.01 *versus* with vehicle control.

Imaging revealed that alternol treatment altered cell morphology and induced cell apoptosis in dose‐dependent manner after 12 hrs (Fig. 2A and B). Those observations were further verified by Annexin V‐FITC/PI staining of cells treated with the indicated concentrations of alternol. As shown in Figure 2C and D, alternol‐induced significant apoptosis in a dose‐dependent manner in both 143B and MG63 cells. To investigate the underlying mechanism, we attempted to explore the pathways involved. As shown in Figure 2, alternol markedly activated caspase‐3 and ‐8 and PARP cleavage in a dose‐dependent manner (Fig. 2E and F), and time‐dependent (Fig. 2G). To further confirm these findings, we investigated the roles of caspase in apoptosis using a total caspase inhibitor z‐VAD‐FMK. As expected, z‐VAD‐FMK inhibited the cell death induced by alternol treatment (Fig. 2H). These data imply that alternol induces dose‐ and time‐dependent cell death *via* caspase‐dependent cell apoptosis.

### Alternol suppresses OS cell migration and invasion *in vitro*

**Figure 3:**
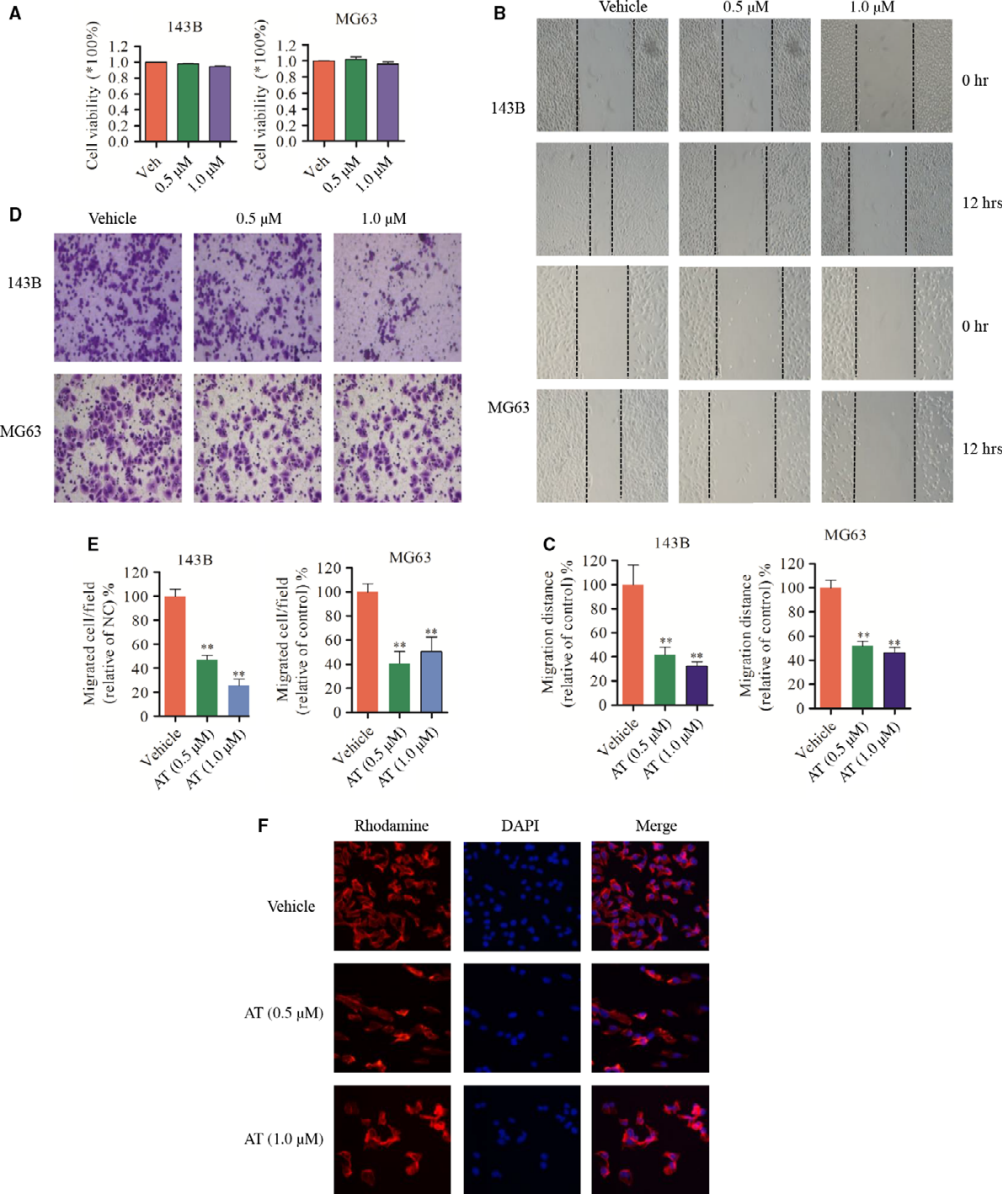
Alternol suppresses OS cell migration and invasion *in vitro* (**A**) 143B and MG63 OS cells were treated with low‐dose alternol (0.5 and 1.0 μM) for 12 hrs, cell viability were measured by CCK8 assay. (**B** and **C**) 143B and MG63 cells were seeded in 12‐well plates. ‘wound’ were created after the cells grow into 80–90% confluence. Cells were treated with alternol (0.5 and 1.0 μM) or vehicle and were photographed in 0 and 12 hrs. Dotted lines indicate the area occupied by the initial scraping and migrated cells were quantified by manual counting. (**D** and **E**) Tumour cells were seeded in the upper chamber of transwell with serum‐free medium, and then alternol (0.5 and 1.0 μM) were added to both chambers. After about 12 hrs, migrated cells were fixed, stained and photographed. Migrated cells were quantified by manual counting. (**F**) Alternol disturbs OS cell actin polymerization. 143B cells were seeded in 12‐well plate and treated with vehicle or alternol (0.5 and 1.0 μM) for 12 hrs, after rhodamine and DAPI staining, images were collected with fluorescence microscope. *P < 0.05 *versus* with vehicle control, **P < 0.01 *versus* with vehicle control.

Pulmonary metastasis is one of the most important malignant phenotypes of OS, and contributes to most patient deaths 23. We further evaluated the ability of alternol to inhibit OS cell migration using 143B and MG63 cells. As shown in Figure 3, low‐dose alternol inhibited OS cell migration (Fig. 3B and C) and invasion (Fig. 3E and D) without significant cell toxicity (Fig. 3A) after 12‐hr treatment. Further cytoskeleton actin staining with rhodamine phalloidin indicated that low‐dose alternol treatment inhibited migration and invasion by reducing OS F‐actin polymerization in 143B cells (Fig. 3F).

### Alternol suppresses the constitutive activation of STAT3 and expression of its target genes in 143B and MG63 cells

**Figure 4:**
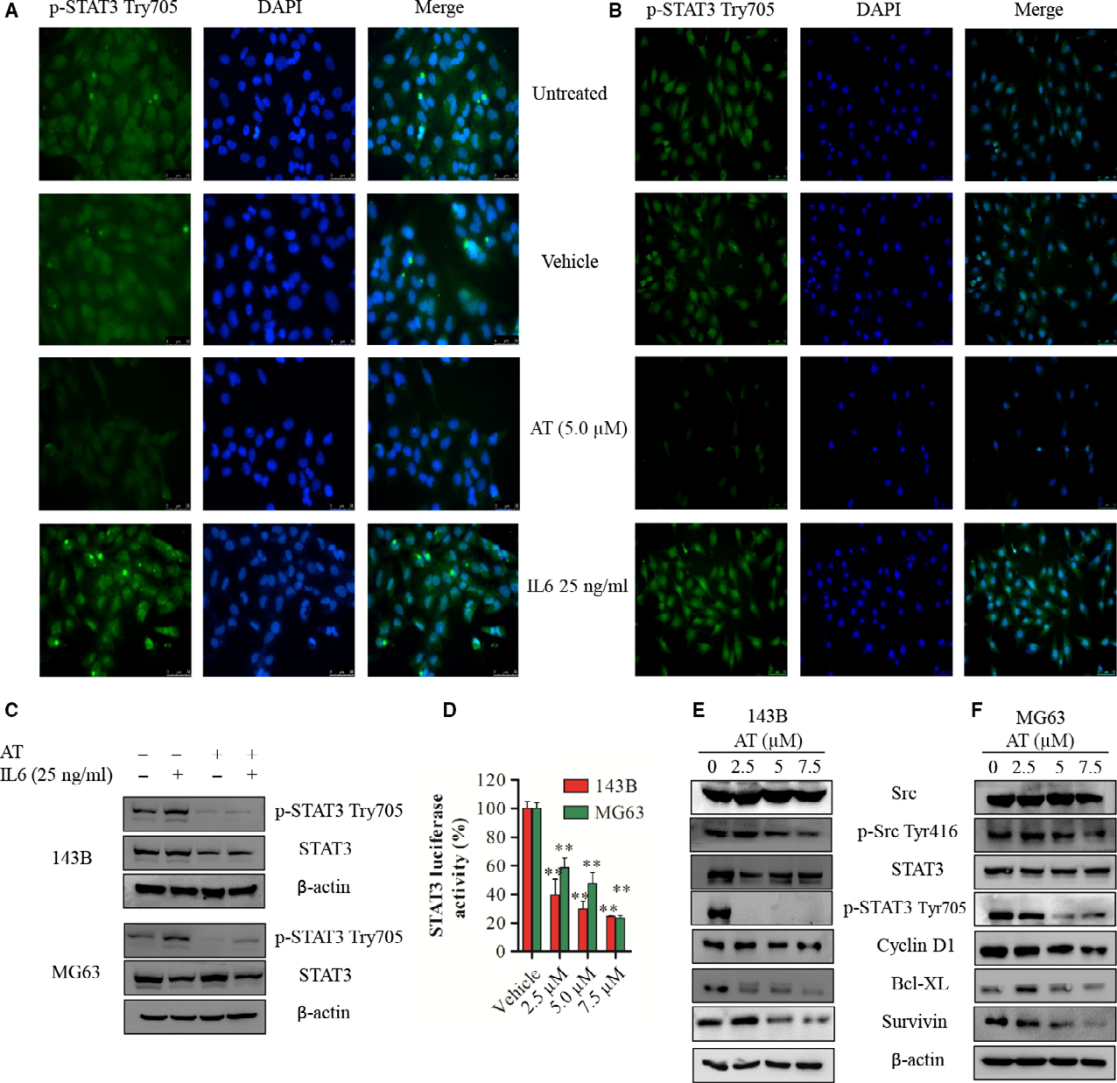
Alternol inhibits the constitutive activation of STAT3 and the expression of its gene products in 143B and MG63 cells (**A** and **B**) 143B and MG63 cells were treated with 5 μM of alternol for 12 hrs or followed by 25 ng/ml of IL‐6 for 30 min. After treatment, the localization of phosph‐STAT3 was analysed by immunofluorescence. STAT3 and phosph‐STAT3 protein expression were measured by immunoblotting (**C**). (**D**) 143B and MG63 cells were transfected with pGMSTAT3‐Lu for 24 hrs and were then incubated with vehicle control or indicated alternol for 12 hrs and STAT3 activity was measured using the STAT3‐dependent dual luciferase assay kit. (**E** and **F**) 143B and MG63 cells were treated with vehicle or indicated alternol for 12 hrs, the expression of STAT3, phosph‐STAT3, Src, phosph‐Src, cyclin D1, Bcl‐xl and survivin were detected by immunoblotting. *P < 0.05 *versus* with vehicle control, **P < 0.01 *versus* with vehicle control.

After incubation for 12 hrs with the indicated dose of alternol, dose‐dependent decreases in STAT3 dual luciferase activity were observed in both 143B and MG63 OS cell using a STAT3‐dependent (pGMSTAT3‐Lu) dual luciferase assay (Fig. 4C). Thus, alternol treatment significantly inhibited STAT3 phosphorylation. Interleukin‐6 is one of the most important inflammatory factors inducing STAT3 phosphorylation at Tyr705. Alternol suppressed IL‐6‐induced STAT3 phosphorylation according to western blot analysis and immunofluorescence (Fig. 4A, B and D). Alternol decreased STAT3 Tyr705 phosphorylation in 143B and MG63 cells in a dose‐dependent manner, and the trends in the changes in Src and phosph‐SrcTyr416 expression were consistent with the changes in STAT3 and phoph‐STAT3 expression after the same alternol treatment. The STAT3 target gene Bcl‐xl, survivin and cyclin D1 were also down‐regulated by alternol treatment by western blot (Fig. 4E and F). These results indicated that alternol inhibits the STAT3 pathway and has a potential as a potent inhibitor of STAT3 signalling in OS research and treatment.

### Alternol triggers ROS generation and activates MAPK pathway in human OS cells

**Figure 5:**
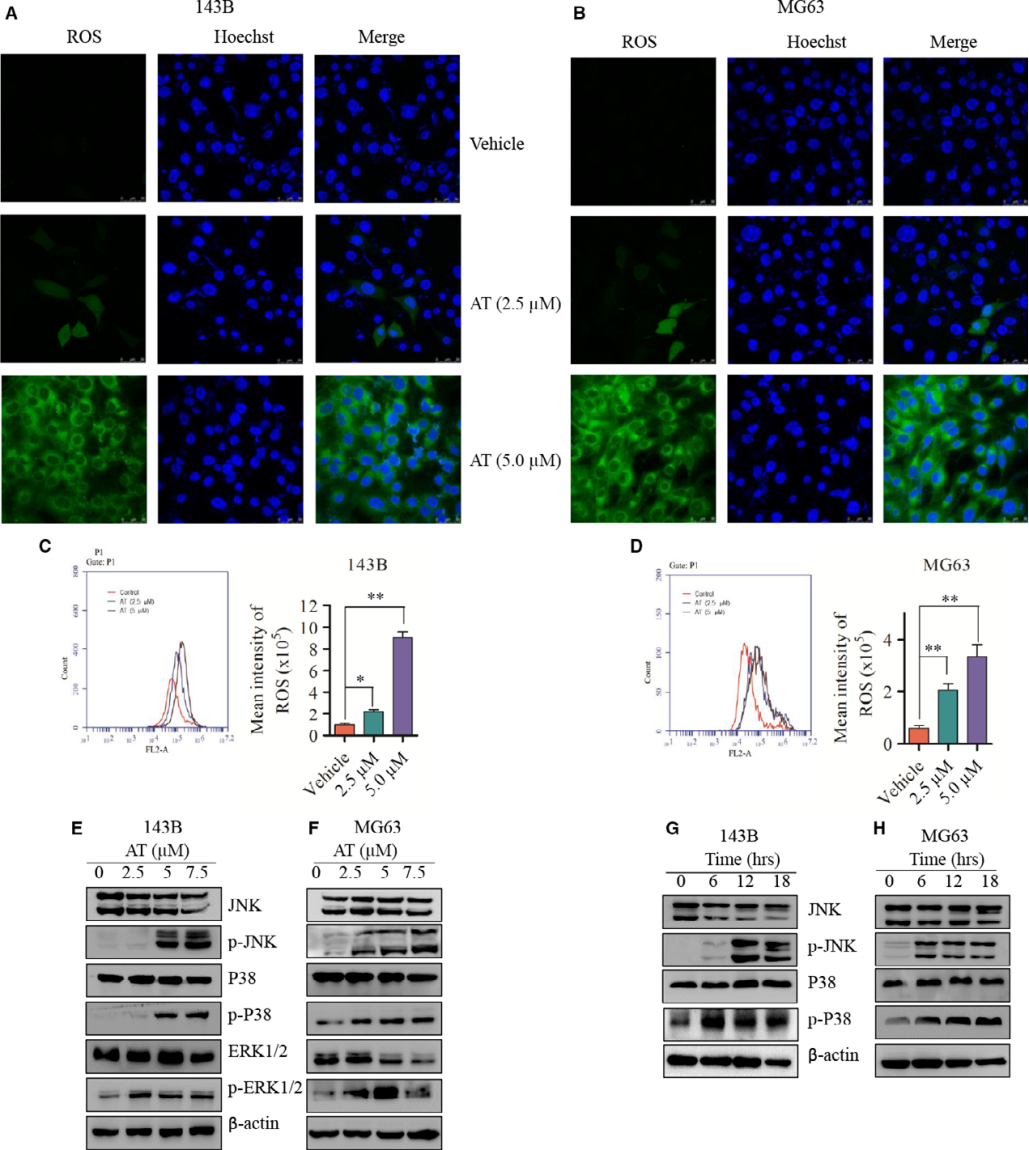
Alternol triggers ROS generation and activates the MAPK pathway in human OS cells. (**A** and **B**) 143B and MG63 cells were treated with vehicle or alternol for 12 hrs and then incubated with DCFH‐DA for 30 min. The ROS generation was measured by confocal microscopy (**A** and **B**) and flow cytometry (**C** and **D**). Representative images were presented. Quantitative analysis of ROS generation was shown in diagrams. (**E** and **F**) 143B and MG63 cells were incubated with alternol for 12 hrs, the expression of SAPK/JNK, phosph‐SAJK/JNK, ERK1/2, phosph‐ERK1/2, p38MAPK, phosph‐p38MAPK were determined by western blot. (**G** and **H**) 143B and MG63 cells were treated with 5 μM alternol for 0, 6, 12, 18 hrs, the expression of SAPK/JNK, phosph‐SAJK/JNK, p38MAPK, phosph‐p38MAPK of were determined by western blot. *P < 0.05 *versus* with vehicle control, **P < 0.01 *versus* with vehicle control.

The accumulation of intracellular ROS is as an important factor in stress‐induced cell death. Treatment with alternol (2.5 and 5.0 μM) for 12 hrs generated ROS in a dose‐dependent manner as revealed by confocal fluorescence microscopic (Fig. 5A and B) and flow cytometry (Fig. 5C and D) with DCF‐DA staining. Western blot assay indicated that the MAPK pathways were activated and up‐regulated in both a time‐ and dose‐dependent manner. Phosph‐SAPK/JNK and phosph‐p38 were notably up‐regulated in alternol‐treated 143B and MG63 cells, whereas phosph‐ERK1/2 was up‐regulated in a dose‐ but not time‐dependent treatment, as shown in Figure 5 E–H (phosph‐ERK1/2 change in the time‐dependent group are not shown). Those data suggest that alternol can trigger ROS generation in human OS cells and activate the MAPK pathway.

### ROS is required for alternol‐induced cell death and G2/M cell cycle arrest

**Figure 6:**
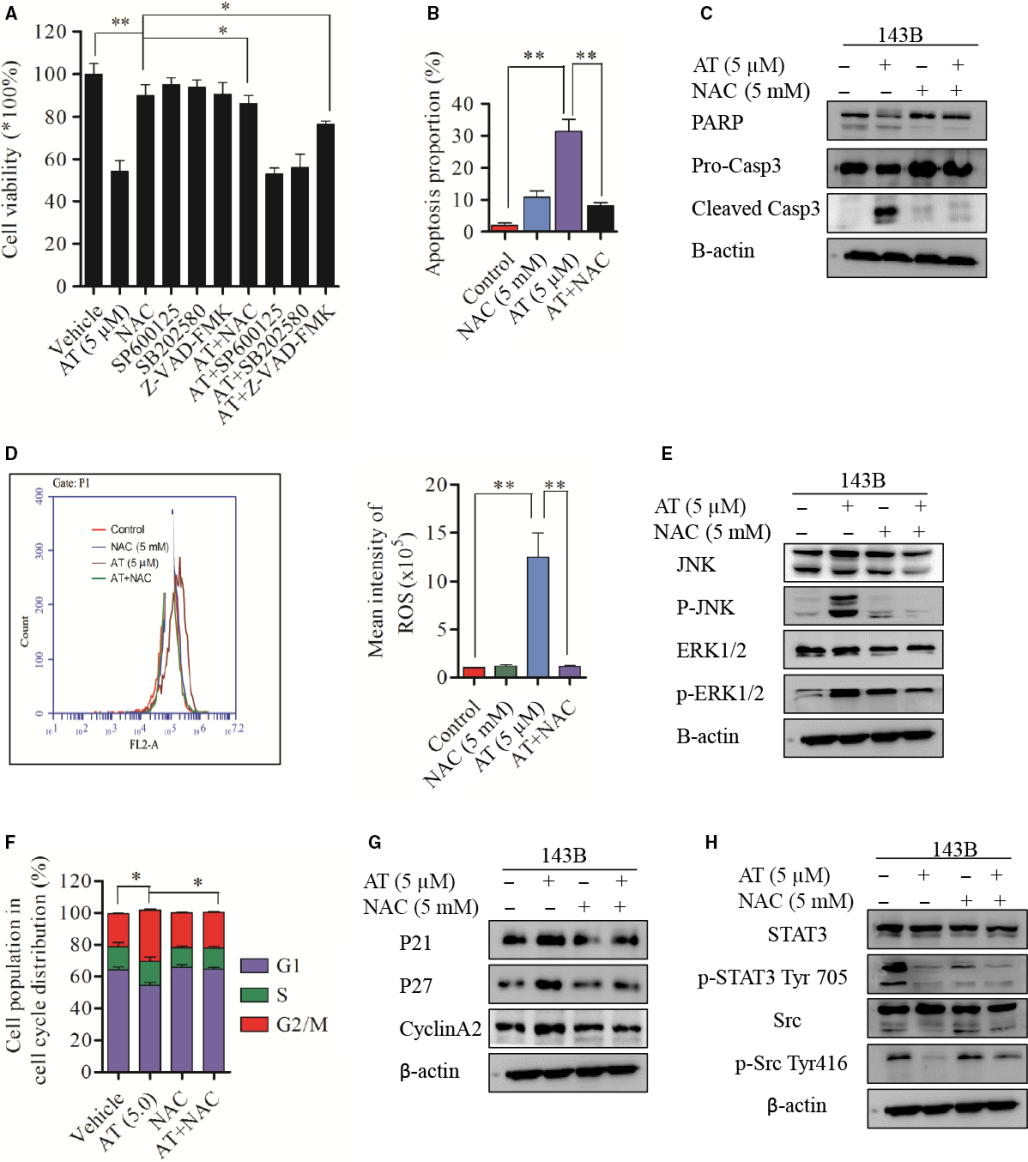
ROS is required for alternol‐induced cell death and G2/M cell cycle arrest. (**A**) 143B cells were incubated with vehicle or alternol with or without presence of NAC, SP600125 and SB202580 for 12 hrs, cell viability was determined by CCK8. 143B cells were incubated with vehicle or alternol with or without presence of NAC for 12 hrs, (**B**) cell apoptosis were analysed by flow cytometry, (**C**) Caspase‐3 and PARP expression were determined with western blot. (**D**) ROS generation of 143B cells was determined by flow cytometry. (**E**) SAPK/JNK, phosph‐SAPK/JNK, ERK1/2, phosph‐ERK1/2 expression were determined with western blot. (**F**) Cell cycles of 143B cells were analysed by flow cytometry. (**G**) P21, p27, cyclinA2 expression was measured by western blot. (**H**) STAT3, phosph‐STAT3 Tyr705, Src, phosph‐Src Tyr416 expression was determined by western blot. *P < 0.05 *versus* with vehicle control, **P < 0.01 *versus* with vehicle control.

To investigate the mechanism underlying the relationship among ROS generation, MAPK activation and alternol‐induced cell death, we treated 143B OS cells with the ROS scavenger NAC, JNK‐specific inhibitor SP600125, the p38MAPK inhibitor SB202580 and global caspase inhibitor Z‐VAD‐FMK in the presence or absence of alternol (5 μM) in CCK8 cell viability assays. When treated with 5 μM alternol in the presence of JNK‐specific inhibitor SP600125 or the p38MAPK inhibitor SB202580, no significant increase in cell viability was observed compared with alternol treatment, whereas either Z‐VAD‐FMK or NAC significantly rescued the alternol‐induced cell death to more than 75% cell survival (Fig. 6A). Next, we confirmed the anti‐apoptotic effect of NAC in alternol‐induced cell death by flow cytometry and western blot. NAC significantly reduced alternol‐induced cell apoptosis compared with alternol treatment. The apoptosis proportion was 27% for alternol group and 9% for alternol combined with NAC group after 12 hrs treatment (Fig. 6B and C). Moreover, the activation of cleaved caspase‐3 and PARP by alternol treatment was rescued by NAC treatment.

We then verified the role of NAC in inhibiting ROS generation using flow cytometry and a western blot assay; ROS generation was inhibited by mean of 1.2 ± 0.2 fold compared to the control group when cells were treated with alternol in the presence of NAC, whereas 5 μM alternol treatment for 12 hrs notably increased ROS generation by 12.5 ± 2.56 fold compared to the vehicle group (Fig. 6D). Western blot analysis revealed that the phosphorylation of JNK and ERK1/2 was significantly increased after alternol treatment and that this can be eliminated in the presence of NAC (Fig. 6E).

Moreover, we observed that NAC treatment exerted a rescue effect on G2/M phase cell cycle arrest induced by alternol treatment, as assessed by cell cycle flow cytometry and western blot. Alternol with NAC reduced G2/M phase proportion (Fig. 6F), and down‐regulated p21 and p27 expression levels compared with alternol treatment (Fig. 6G). These data indicate that NAC treatment can rescue alternol‐induced ROS generation and cell apoptosis, and reverse the G2/M cell cycle arrest in OS cells.

We next further verified the involvement of ROS in alternol‐induced STAT3 inactivation, western blot analysis demonstrated that the ROS scavenger NAC had no significant rescue effect on phosph‐STAT3 inactivation but instead restored the decrease in phoph‐Src Tyr416 (Fig. 6H). These data indicate that ROS may not be pivotal for suppression of constitutive STAT3 in alternol treatment.

### Alternol inhibits the growth of OS *in vivo*

**Figure 7:**
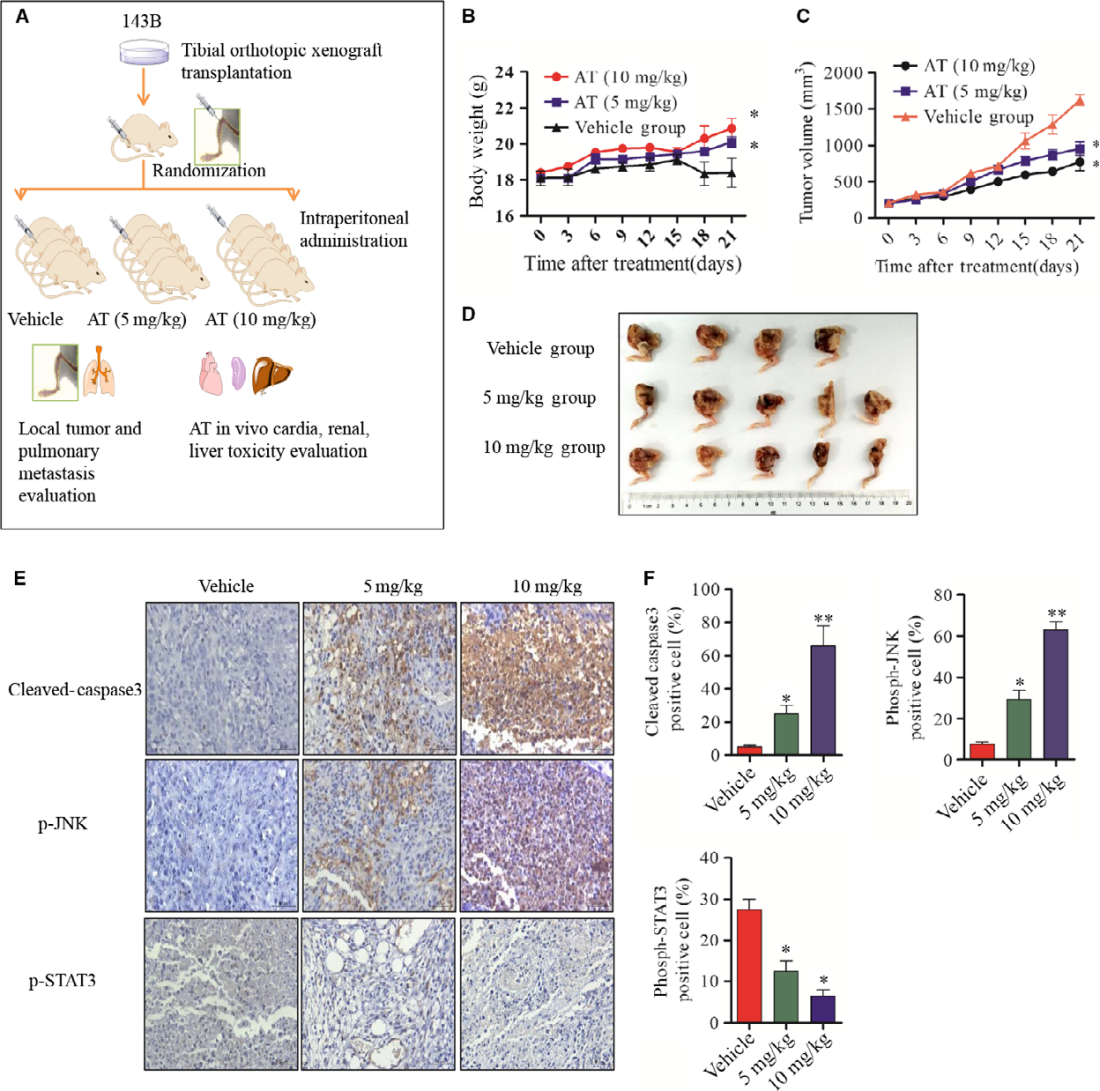
Alternol inhibits the growth of OS *in vivo* (**A**) Flow diagram of *in vivo* nude mouse tibia orthotopic model establishment and treatment plan. (**B** and **C**) Body weight and tumour size were measured every 3 days. (**D**) Macroscopic appearance of tibia primary osteosarcoma tumours in BABL/c nude mice after treatment with alternol or vehicle. (**E** and **F**) The expression of cleaved caspase‐3, phosph‐JNK, phosph‐STAT3 were examined by immunohistochemistry using tibia tumour samples slides. *P < 0.05 *versus* with vehicle control, **P < 0.01 *versus* with vehicle control.

The *in vivo* anti‐tumour effect of alternol was determined *via* ip administration of alternol in nude mice bearing 143B cells in the tibia (Fig. 7A and D). Alternol treatment at doses of 5 and 10 mg/kg resulted in significant decrease in tumour volume of 43.8% and 60%, respectively, after the drug was administrated eight times (Fig. 7C). Notably, 5 and 10 mg/kg alternol treatment induced 5.8% and 14.4% weight loss in mice respectively (Fig. 7B). An immunohistochemistry assay revealed an increase in the mean positive areas for cleaved caspase‐3 and phosph‐JNK and decrease in phospho‐STAT3 in alternol‐treated tumour tissues (Fig. 6F). These results demonstrated that alternol can inhibit the *in vivo* growth of orthotopic OS in nude mice.

## Discussion

In this study, we investigated the anti‐tumour effect of alternol in human OS cell lines and *in vivo* models. Alternol inhibited cell proliferation, migration and induced G2/M phase cell cycle arrest and caspase‐dependent apoptosis in 143B and MG63 cells. The most interesting finding was that alternol suppressed the phosphorylation of STAT3 and the expression of its target gene products Bcl‐xl, surviving and cyclin D1 in 143B and MG63 cells. In addition, massive intracellular ROS generation with MAPK pathway activation was shown to play a pivotal role in the anti‐tumour effect of alternol in both OS cell lines. The majority of OS cell death induced by alternol treatment was rescued by NAC, a specific scavenger of ROS, and Z‐VAD‐FMK, a general inhibitor of caspase in apoptosis, had a similar protective effect on cell viability. However, inhibitors of SAPK/JNK and MAPKp38 did not attenuate alternol‐induced cell death. Alternol also inhibited orthotopic tibial OS tumour growth *in vivo*.

Usually, STATs are phosphorylated by JAK or Src tyrosine kinase in response to the binding of growth factors or cytokines to their corresponding receptors 24. Our study is the first to report that alternol can inhibit phosphorylation of STAT3 in OS in *in vitro* and *in vivo* preclinical models. In addition, alternol reduced constitutive STAT3 phosphorylation in a dose‐dependent manner, and phosph‐Src tyrosine kinases were down‐regulated in alternol‐treated OS cells, as were the STAT3 target genes Bcl‐xl, survivin and cyclin D1. By contrast, alternol treatment resulted in down‐regulation of the expression of Bcl‐xl, a protein in the Bcl‐2 family, but not Bcl‐2. Bcl‐xl is a member of anti‐apoptotic protein Bcl‐2 family that plays pivotal role in cell apoptosis, whereas cyclin D1 functions as a CDKs regulator, and required for progression through the cell cycle. These data indicated that alternol‐induced cell cycle arrest and cell apoptosis are modulated by inactivation of the STAT3 pathway. Kim *et al*. 25 reported similar STAT3 pathway inhibition by 6‐shogaol, an active ingredients in ginger that can inhibit the constitutive STAT3 signalling cascade, induce ROS‐mediated JNK, p38 MAPK, and ERK1/2 activation, and induce substantial apoptosis *via* the down‐modulation of gene products that mediate tumour cell survival, proliferation, invasion and metastasis in human breast cancer. However, Kim *et al*. did not further observe a correlation between ROS generation and STAT inactivation in their study. Although Kim *et al*. 26 reported an anti‐tumour effect of carnosic acid (CA) in human colon cancer cells, they observed that CA can induce ROS generation and suppress STAT phosphorylation in human colon cancer cell lines. Carnosic acid‐induced STAT inhibition was restored by a ROS scavenger NAC, which is not in agreement with our findings. Kim *et al*. posited that it is likely that CA‐induced ROS cause oxidative modification of the cysteine residues of JAK2, Src or STAT3 proteins. In this study, NAC treatment did not abolish alternol‐induced STAT inactivation, but partially rescued Src phosphorylation in alternol‐treated 143B cells, these data indicated that Src may function as a possible upstream regulator of alternol‐induced STAT3 inhibition.

Excessive intracellular ROS generation, or the failure of oxidant scavenging systems, can disrupt cellular functions by causing oxidation of lipids, proteins and DNA structure stability, eventually resulting in cell death 27. Most preclinical cancer studies 28, 29 have indicated excessive ROS generation can induce cytochrome c releasing from the mitochondria to the cytosol, and lead to caspase‐dependent cell killing. However, others have observed that anti‐oxidants, which are presumed to be exert their effect by squelching ROS and reducing genomic instability, can inhibit several tumour models *in vivo* in a HIF‐dependent manner 30, indicating that ROS generation is a cell death inducer and cell signal agent in cancer. The underlying mechanism remains unclear. In this study, ROS generation influenced alternol‐induced cell apoptosis, G2/M cycle arrest and MAPK phosphorylation and that an ROS scavenger NAC reversed intracellular ROS, cell death and MAPK phosphorylation. Moreover, treatment with a JNK inhibitor or p38MAPK inhibitor treatment did not rescue cells from alternol‐induced OS cell deaths, indicating that MAPK activation is a cellular stress response to ROS generation. The current work focused on ROS generation, MAPK and anti‐tumour effect of alternol. In contrast to the JNK activation mechanism of celastrol in OS reported by Li *et al*. 28, we observed that MAPK (JNK, ERK1/2 and p38) phosphorylation was induced by alternol treatment but that phosphorylation was not critical to cell death. JNK or p38 inhibition did not abate alternol‐induced cell killing. Increased phosphorylation of JNK 31, 32, and p38MAPKs 33 is normally associated with induction of apoptosis, whereas ERK1/2 primarily plays a role in cell proliferation instead of apoptosis 34, Researcher has demonstrated that persistent over‐activation of ERK1/2 induces cell cycle arrest by stabilizing the p21 complex 35. Jung *et al*. 36 (PMID 26212545) observed that sugiol, an anti‐tumour compound for prostate cancer, increases ROS levels, leading to activation of ERK and subsequent inhibits STAT3 activity. In addition, sugiol‐induced STAT3 dephosphorylation at Tyr705 and STAT3 phosphorylation at Ser727 were abrogated by pretreatment with the ERK inhibitor U0126. Furthermore, IP experiment revealed that STAT3 was inactivated in response to sugiol‐induced ERK activation, and this process was induced by the direct interaction between STAT3 and ERK. Therefore, ROS‐induced cell apoptosis and cell cycle arrest may explain the increased phosphorylation of JNK, p38MAPK and ERK1/2, and similar MAPK activation in the study by Li *et al*. 37. The underlying interactions among MAPK pathway activation, ROS generation and STAT3 inhibition in OS cells require further elucidation.

In contrast to the manual planting, self‐synthesis, complete‐synthesis and biological‐synthesis methods used to acquire paclitaxel, alternol can be produced and extracted from epiphyte‐ferment technology and is thus easily accessible and cheaper for clinical application. However, the anti‐tumour ability of alternol is not as well studied as that of paclitaxel. Alternol is considered a promising anti‐tumour treatment for some patients and is also a promising STAT3 inhibitor candidate for the development of anti‐tumour drugs that target OS.

## Conflicts of interest

The authors declare no potential conflicts of interest.
